# Assessment of atrial regional and global electromechanical function by tissue velocity echocardiography: a feasibility study on healthy individuals

**DOI:** 10.1186/1476-7120-3-4

**Published:** 2005-02-18

**Authors:** Miguel Quintana, Peter Lindell, Samir K Saha, Francesca del Furia, Britta Lind, Satish Govind, Lars-Åke Brodin

**Affiliations:** 1 Department of Clinical Physiology, Karolinska University Hospital, Huddinge. The Karolinska Institute, Stockholm, Sweden; 2 Department of Cardiology, Karolinska University Hospital, Huddinge. The Karolinska Institute, Stockholm, Sweden

**Keywords:** atrial mechanical function, atrial electrical impulse spreading, left ventricular function, tissue Doppler echocardiography

## Abstract

**Background:**

The appropriate evaluation of atrial electrical function is only possible by means of invasive electrophysiology techniques, which are expensive and therefore not suitable for widespread use. Mechanical atrial function is mainly determined from atrial volumes and volume-derived indices that are load-dependent, time-consuming and difficult to reproduce because they are observer-dependent.

**Aims:**

To assess the feasibility of tissue velocity echocardiography (TVE) to evaluate atrial electromechanical function in young, healthy volunteers.

**Subjects and methods:**

We studied 37 healthy individuals: 28 men and nine women with a mean age of 29 years (range 20–47). Standard two-dimensional (2-D) and Doppler echocardiograms with superimposed TVE images were performed. Standard echocardiographic images were digitized during three consecutive cardiac cycles in cine-loop format for off-line analysis. Several indices of regional atrial electrical and mechanical function were derived from both 2-D and TVE modalities.

**Results:**

Some TVE-derived variables indirectly reflected the atrial electrical activation that follows the known activation process as revealed by invasive electrophysiology. Regionally, the atrium shows an upward movement of its walls at the region near the atrio-ventricular ring with a reduction of this movement towards the upper levels of the atrial walls. The atrial mechanical function as assessed by several TVE-derived indices was quite similar in all left atrium (LA) walls. However, all such indices were higher in the right (RA) than the LA. There were no correlations between the 2-D- and TVE-derived variables expressing atrial mechanical function. Values of measurement error and repeatability were good for atrial mechanical function, but only acceptable for atrial electrical function.

**Conclusion:**

TVE may provide a simple, easy to obtain, reproducible, repeatable and potentially clinically useful tool for quantifying atrial electromechanical function.

## Introduction

The enlargement of left atrial (LA) diameter is associated with cardiovascular disease and is a risk factor for atrial fibrillation, stroke and death. [1-6] LA function reliably predicts exercise capacity in patients with recent myocardial infarction[7] or non-ischemic dilated cardiomyopathy[8] and differs in patients with ischemic and dilated cardiomyopathy.[9] Moreover, LA volume is an independent prognostic factor in several subsets of patients. [10-12] Although commonly used, LA size assessed by M-mode echocardiography does not correlate well with LA volumes, so several methods to estimate LA volumes have been developed.[13,14] The LA reservoir, conduit and pump functions may be estimated from volume measurements. [15-19] However, the reliability and clinical usefulness of those methods have been poorly studied. Pulsed-wave Doppler interrogation of the blood flow velocity during atrial contraction, the peak mitral inflow A wave, and its velocity time integral have also been used as surrogate markers of atrial function. [20-22] These variables represent the diastolic properties of the LV [23-25] and do not accurately reflect atrial mechanical properties. Tissue velocity echocardiography (TVE) has now been developed as a valuable tool for the evaluation of left and right ventricular systolic and diastolic functions.[26,27] Furthermore, this technique has also been used to assess the regional functions of the left and right atrium.[28,29] Although atrial anatomy was described more than a century ago, a new interest in atrial anatomy and its relations with atrial electromechanical function has only recently emerged.[30,31] Conventionally, atrial electrical function has been evaluated from resting electrocardiography (ECG), and more accurately by invasive electrophysiology techniques. [32-34] The rapid development of these invasive techniques has improved not only diagnostic capabilities,[35,36] but also our understanding of how the electrical impulse spreads through atrial tissues,[37,38] and has led to improvements in the treatment of supra-ventricular tachy-arrhythmias. [38-40] However, the invasive nature and the high costs of these procedures limit their widespread use and repeatability. Therefore, the development of noninvasive, safe, accurate and repeatable methods that might provide similar information is necessary. We aimed here to find simple and repeatable methods to assess both electrical and mechanical regional atrial functions by means of TVE.

## Methods

### Population

We studied 37 healthy individuals: 28 men and nine women with a mean age of 29 years (range 20–47). The individuals were recruited from among hospital employees, cardiovascular technicians and medical students. None showed symptoms of cardiovascular disorders or were receiving pharmacological cardiovascular agents. All had normal standard two-dimensional (2-D) and Doppler echocardiograms. All subjects were on sinus rhythm and none hade A-V or intra-ventricular conduction defects. The Ethical Committee at the Karolinska University Hospital, Huddinge, approved the study. All individuals received written information and gave informed consent.

### Echocardiography

A standard 2-D and Doppler echocardiogram with superimposed TVE images was performed using a 3.5 MHz transducer with commercially available equipment (System FiVe™, GE Vingmed, Horton, Norway). Standard parasternal short- and long-axis views as well as apical 2-, 3- and 4-chamber views acquired at expiratory apnea with at least 90 frames per second were digitized during three consecutive cardiac cycles in cine-loop format for off-line analysis.

### Off-line analyses

All echocardiographic images were analyzed off-line using software (Echopac™ 6.3.4, GE Vingmed) for the calculation of standard 2-D and Doppler echocardiography as well as for the analysis of TVE variables.

### Standard 2-D and Doppler echocardiography

Measurements of the left ventricular (LV) function comprised septum and posterior wall thickness; LV end-systolic and diastolic dimensions; LV fractional shortening, and LV ejection fraction (LVEF) according to international standards.[41] Measurements of atrial function comprised left atrial (LA) diameter measured from the parasternal long axis; right atrium (RA) and LA long and short axes; LA and RA maximal volume; LA and RA minimal volume, and RA and LA volumes at the beginning of the P-wave measured from the apical 4- and 2-chamber views. LA and RA ejection fractions were measured according to the formula: (maximal volume-minimal volume)/maximal volume. LA and RA active emptying values were calculated as (volume at P-wave-minimal volume)/volume at P-wave.[8,9,42]

### Tissue velocity echocardiography

**Figure 1:**
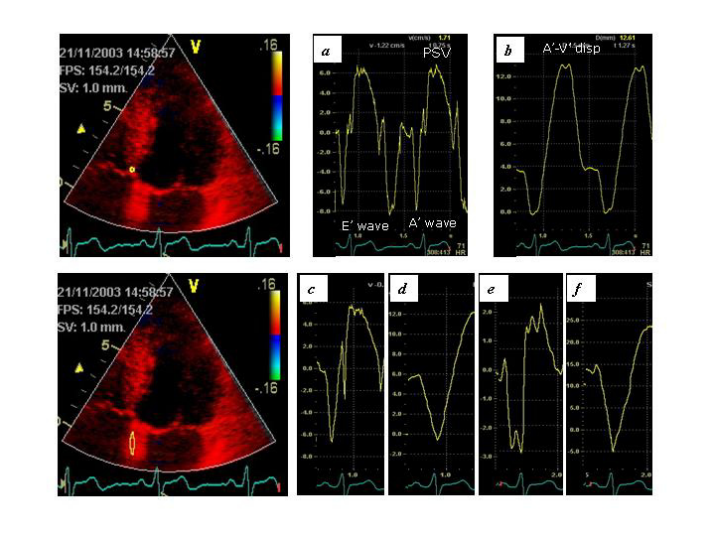
Assessment of atrial and ventricular mechanical function. The upper panel shows the systolic and diastolic velocities (a) and the A-V place displacement (b) measured at the basal level of the inter ventricular septum. The lower panel shows the atrial velocity (c), atrial displacement (d), atrial strain rate (e) and atrial strain (f) measured at the inter atrial septum below the mitral ring.

The RV and LV long axis functions were assessed from apical views. Six basal LV segments were identified as follows: the RV free wall; the LV postero-septal wall, and the LV lateral wall from the apical 4-chamber view; the LV inferior and anterior walls from the apical 2-chamber view, and the LV posterior wall from the apical 3-chamber view. A sample volume was positioned at the base of each ventricular wall excluding the A-V plane during the entire heart cycle to obtain a tissue velocity profile during three consecutive cardiac cycles. Both systolic and diastolic phases of the velocity profile were considered and the following parameters were analyzed (upper part of Fig. 1): *peak systolic velocity *(PSV, in centimeters per second), measured at the peak velocity during the ejection period; *peak velocity at early diastole (*E'-wave, in centimeters per second), measured at the peak velocity at early diastole, and *peak velocity at late diastole *(A'-wave, in centimeters per second), measured at the peak velocity at late diastole. The *atrio-ventricular myocardial wall displacement *(A'-V' disp., in millimeters) in the long axis was obtained by automated temporal integration of the PSV of the basal segments during the ejection period.

**Figure 2:**
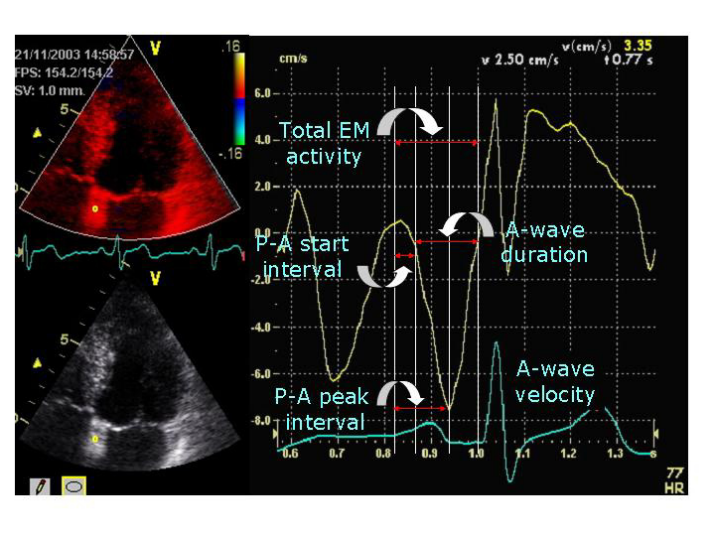
Assessment of some time intervals and Aa' wave velocity at the low level of the inter atrial septum

The different atrial walls were identified from the same apical views as follows: the right atrial wall (RA), the inter-atrial septum (IAS), and the left atrial lateral wall (LA-Lat) from the apical 4-chamber view; the left atrial inferior wall (LA-Inf) and the left atrial anterior wall (LA-Ant) from the apical 2-chamber view; and the left atrial posterior wall (LA-Post) from the apical 3-chamber view. Each atrial wall was studied at low and mid levels, placing a 2 mm sample volume at low atrial walls excluding the A-V plane during the entire cardiac cycle and at the mid portion of each atrial wall. The regional electromechanical function at each atrial wall was studied by the following time intervals (Figure 2). The *PA-start interval *(P-Aa' start) was defined as the time between the beginning of the P-wave on the monitor's ECG to the start of the A' wave on the TVE-curve profile. The *PA-peak interval *(P-Aa' peak) was the time between the beginning of the P-wave on the monitor's ECG to the peak of the A' wave on the TVE-curve profile. The *A-wave duration *(Aa'-dur.) was the time from the beginning to the end of the A'-wave on the TVE-curve profile. The *total electromechanical activity *(TEMA) was the time between the beginnings of the P-wave on the monitor ECG to the end of the A' wave on the TVE-curve profile.

The regional mechanical function of each atrial wall was assessed by the *peak velocity during atrial contraction *(Aa' peak vel.), the *atrial displacement occurring during atrial contraction *(Aa' disp.) and the *ratio of atrial displacement measured at atrial level to the total LV myocardial displacement measured at ventricular level *(Aa' cont.) (lower part of Figure 1). In addition, *strain rate *(Aa' SR) and *strain *(Aa' S) were assessed in each low atrial wall using a sample volume of 12 mm.

### Statistical analyses

Data are presented as means ± standard deviations (SD). Analysis of variance (ANOVA) with repeated measures was used to test statistical significance of the studied variables at different atrial and ventricular walls. When ANOVA showed statistically significant differences among atrial and ventricular walls, post hoc analysis with Bonferroni's test was performed to assess differences among those walls. Correlation coefficients were calculated to assess the relationship among several markers of atrial mechanical function. The inter- and intra-observer repeatability and measurement errors for variables reflecting the atrial electromechanical function were assessed by the coefficient of variation and by the British Standards Institution method, the value below which the difference between two measurements will lie with a probability of 0.95. *P *< 0.05 was considered statistically significant.

## Results

All demographic features and measures of standard 2-D and pulsed-wave Doppler echocardiography data are shown in Table 1. Of interest, no differences were found between measures of RA and LA functions, as assessed by short or long axes, or among volumes and volume-derived indices. TVE-derived variables assessing the RV and LV long-axis systolic and diastolic functions are shown in Table 2. No significant differences among LV walls were found for any index of systolic and diastolic function. TVE-derived variables obtained from the RV free wall were significantly different from each LV wall.

**Table 1:** Demographic features and resting echocardiographic data. Numbers are means ± SD.

Age, years	29 ± 7		
Gender (M/F)	28/9		
Height, cm	175 ± 8		
Weight, kg	76 ± 14		
Heart rate, bpm	66 ± 12		
P-Q time, ms	166 ± 16		
LA diameter, mm/m^2^	18.9 ± 1.5		
Septal wall thickness, mm	9.6 ± 1.1		
Posterior wall thickness, mm	9.5 ± 1.2		
LV end diastolic diameter, mm/m^2^	26.5 ± 2.3		
LV fractional shortening, %	35 ± 6		
LV ejection fraction, %	72 ± 8		
E-wave, cm	90 ± 17		
A-wave, cm	56 ± 12		
E/A ratio	1.68 ± 0.45		
		
			
LA long axis, mm/m^2^	26 ± 2	RA long axis, mm/m^2^	25 ± 3
LA short axis, mm/m^2^	21 ± 3	RA short axis, mm/m^2^	22 ± 2
LA maximal volume, ml/m^2^	29 ± 5	RA maximal volume, ml/m^2^	31 ± 7
LA minimal volume, ml/m^2^	15 ± 3	RA minimal volume, ml/m^2^	17 ± 4
LA P-wave volume, ml/m^2^	18 ± 4	RA P-wave volume, ml/m^2^	19 ± 5
LA ejection fraction, %	49 ± 9	RA ejection fraction, %	46 ± 10
LA active emptying, %	17 ± 7	RA active emptying, %	15 ± 9

**Table 2:** Systolic and diastolic myocardial velocities measured at different right and left ventricular walls

**Variable**	**Ventricular walls**					
	**RV**	**Post-sep**	**Lateral**	**Inferior**	**Anterior**	**Posterior**

**PSV, cm/s**	10.5 ± 1.3	6.8 ± 0.9	8.5 ± 1.8	7.5 ± 1.1	7.9 ± 1.6	7.6 ± 1.3
**E'-wave, cm/s**	10.2 ± 2.3	9.9 ± 1.1	12.2 ± 1.5	10.6 ± 2.1	10.1 ± 2.1	12.4 ± 1.9
**A'-wave, cm/s**	8.4 ± 2.8	5.9 ± 1.3	4.7 ± 1.5	6.3 ± 2.0	4.7 ± 1.7	6.1 ± 2.1
**E'/A' ratio**	1.2 ± 0.1	1.7 ± 0.1	2.6 ± 0.1	1.7 ± 0.2	2.1 ± 0.2	2.0 ± 0.1
**A'-V' disp., mm**	21.5 ± 3.5	13.6 ± 1.5	13.7 ± 2.0	15.3 ± 1.6	13.7 ± 1.9	15.4 ± 1.8
**Atrial disp., mm**	5.8 ± 2.2	4.1 ± 1.3	2.5 ± 0.8	3.8 ± 1.5	2.9 ± 0.9	3.0 ± 1.1
**Atrial cont., %**	27 ± 8	30 ± 9	19 ± 7	25 ± 9	22 ± 8	20 ± 6

Abbreviations: cont., contribution; disp., displacement; PSV, peak systolic velocity

**Figure 3:**
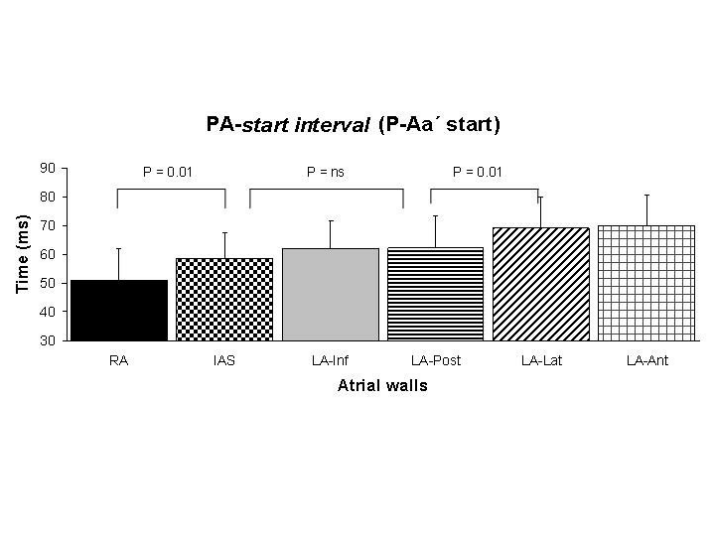
Assessment of the duration of the *PA-start interval *in all atrial walls. Comparisons were done with ANOVA with repeated measures and the Bonferroni's test.

**Figure 4:**
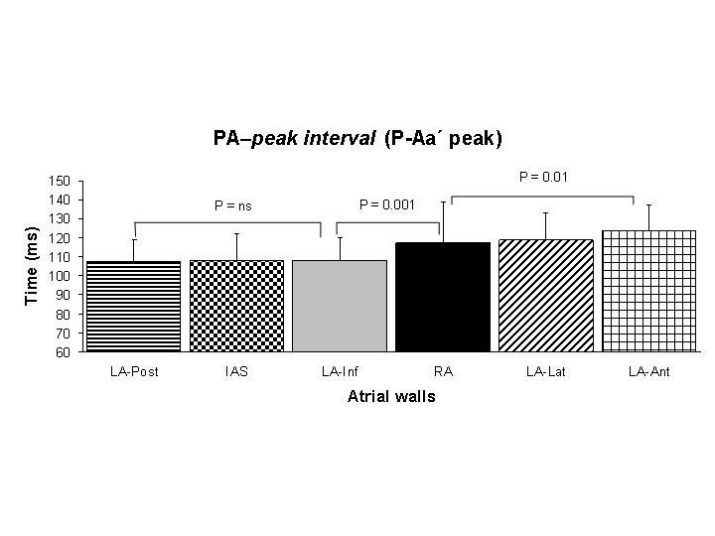
Assessment of the duration of the *PA-peak interval *in all atrial walls. Comparisons were done with ANOVA with repeated measures and the Bonferroni's test.

Table 3 shows several time intervals. The *PA-start interval *(P-Aa' start) was longer at low atrial levels in each atrial wall than at the mid atrial level and shorter at the RA than for all LA walls (Fig. 3). Some statistical significant differences among different LA walls were also found. The *PA-peak interval *(P-Aa' peak) was similar at low and mid atrial levels in almost all atrial walls with exceptions in the inferior and posterior LA walls. This interval was shorter for the IAS, inferior and posterior LA walls than for the lateral and anterior LA walls at mid and low levels (Fig. 4). The *A-wave duration *(Aa'-dur.) was shorter at the low atrial level than at the mid atrial level in each atrial wall, but not in the inferior and posterior LA walls. The *total electromechanical activity *(TEMA) was similar in all RA and LA atrial walls measured at low and mid levels, and no differences were found between any of the LA walls.

**Table 3:** Time intervals expressed in milliseconds measured at low and mid atrial levels in the myocardial walls of the RA and LA.

**Variables**	***Level***	**RA**	**IAS**	**LA-Lat**	**LA-Inf**	**LA-Ant**	**LA-Post**	P*
**P-Aa' start**	*Low*	51 ± 11	59 ± 9	69 ± 11	62 ± 10	70 ± 10	62 ± 11	< 0.001
**(ms)**	*Mid*	38 ± 9	47 ± 8	57 ± 9	51 ± 11	59 ± 10	52 ± 11	< 0.001
	***P***	< 0.001	< 0.001	< 0.001	< 0.001	< 0.001	< 0.001	
**P-Aa' peak**	*Low*	117 ± 22	108 ± 14	119 ± 14	108 ± 12	123 ± 14	107 ± 11	<0.001
**(ms)**	*Mid*	110 ± 20	104 ± 15	115 ± 15	98 ± 17	123 ± 16	99 ± 17	< 0.01
	***P***	0.06	0.04	0.1	< 0.001	0.9	< 0.001	
**Aa'-dur.**	*Low*	135 ± 16	120 ± 16	106 ± 10	118 ± 13	113 ± 12	112 ± 12	< 0.001
**(ms)**	*Mid*	145 ± 19	127 ± 16	117 ± 10	115 ± 12	121 ± 16	115 ± 12	< 0.01
	***P***	< 0.001	< 0.001	< 0.001	0.1	< 0.001	0.07	
**TEMA**	*Low*	186 ± 17	179 ± 18	175 ± 15	179 ± 15	183 ± 14	174 ± 12	0.07
**(ms)**	*Mid*	183 ± 21	174 ± 16	173 ± 15	175 ± 15	180 ± 19	167 ± 17	0.2
	***P***	0.3	0.1	0.5	0.1	0.3	0.06	

**Abbreviations**: Aa'-dur., duration of the A wave; IAS, inter-atrial septum; LA-Ant, left atrial anterior wall; LA-Inf, inferior left atrial wall; LA-Lat, left lateral atrial wall; LA-Post, left posterior atrial wall; ms, milliseconds; **P**, by paired *t *test; P*, by analysis of variance; P-Aa' start, time from the beginning of the P-wave to the start of the A-wave; P-Aa' peak, time from the beginning of the P-wave to the peak of the A-wave; RA, right atrial wall; TEMA, total electromechanical activity.

**P-Aa' start Low**: RA *vs *all LA-walls (*P *< 0.001), IAS and LA-Inf *vs *LA-Lat (*P *< 0.001)

**P-Aa' start Mid**: RA *vs *all LA-walls (*P *< 0.01), IAS *vs *LA-Lat and LA-Ant (*P *< 0.001); LA-Inf *vs *LA-Lat and LA-Inf (*P *< 0.001); LA-Post *vs *LA-Ant (*P *< 0.01)

**P-A'a peak Low and Mid**: IAS, LA-Inf and LA-Post *vs *LA-Lat and LA-Ant (*P *< 0.001 for all comparisons)

**Aa' dur. Low**: RA, IAS and LA-Inf *vs *LA-Lat (*P *< 0.001)

**Aa' dur. Mid**: RA *vs *all walls (*P *< 0.001), IAS *vs *LA-Lat, LA-Inf and LA-Post (*P *< 001)

**TEMA Low**: RA *vs *LA-Lat, LA-Inf and LA-Post (*P *< 0.001). No differences among all LA-walls.

**TEMA Mid**: RA *vs *LA-Lat, LA-Inf, LA-Post (*P *< 0.001), LA-Ant *vs *LA-Inf and LA-Post (*P *< 0.001)

Table 4 shows several velocities and velocity-derived variables: *The peak velocity during atrial contraction *(Aa' peak vel.) was higher at low than mid levels in each atrial wall, but no significant differences were found between any LA walls. This variable was higher in RA than in all LA walls. Similar results were found for the *atrial displacement occurring during atrial contraction *(Aa' disp.) and the *ratio of atrial displacement measured at atrial level to the total LV myocardial displacement measured at ventricular level *(Aa' cont.). The *strain rate *(Aa' SR) was higher in the RA than in all LA-walls, and lower in the IAS than in the lateral and posterior LA walls. The *strain *(Aa' S) was higher in the RA than in all LA-walls, and no differences were found between LA walls.

**Table 4:** Myocardial velocity and velocity-derived variables measured at right and left atrial myocardial walls.

**Variables**	***Level***	**RA**	**IAS**	**LA-Lat**	**LA-Inf**	**LA-Ant**	**LA-Post**	P*
**Aa' peak vel.**	*Low*	8.1 ± 2.7	6.3 ± 1.4	6.2 ± 1.7	6.9 ± 1.8	6.3 ± 1.8	6.8 ± 1.8	NS
**(cm/s)**	*Mid*	6.9 ± 2.4	5.2 ± 1.5	5.7 ± 1.4	5.1 ± 1.7	5.7 ± 1.8	5.4 ± 1.7	NS
	***P***	< 0.001	< 0.001	< 0.001	< 0.001	< 0.01	< 0.001	
**Aa' disp.**	*Low*	6.7 ± 2.3	4.5 ± 0.9	3.7 ± 1.0	4.2 ± 1.0	4.1 ± 1.2	4.0 ± 0.9	NS
**(mm)**	*Mid*	5.8 ± 2.5	3.2 ± 0.9	3.4 ± 0.9	2.9 ± 0.7	3.1 ± 1.3	3.0 ± 0.8	NS
	***P***	< 0.001	< 0.001	< 0.001	< 0.001	< 0.001	< 0.001	
**Aa' cont.**	*Low*	31 ± 10	33 ± 8	29 ± 10	28 ± 7	31 ± 12	26 ± 6	NS
**(%)**	*Mid*	27 ± 11	24 ± 7	25 ± 9	19 ± 6	23 ± 11	19 ± 5	NS
	***P***	< 0.001	< 0.001	0.02	< 0.001	< 0.001	< 0.001	
**Aa' SR (s-^1^)**		-4.9 ± 0.8	-2.7 ± 0.7	-3.7 ± 0.9	-3.2 ± 0.8	-3.3 ± 1.0	-3.7 ± 1.0	0.001
**Aa' S (%)**		29 ± 7	14 ± 5	15 ± 4	16 ± 5	15 ± 6	16 ± 4	NS

**Abbreviations**: Aa' cont., atrial contribution; Aa' disp., atrial displacement; Aa' peak vel., atrial A-wave peak velocity; Aa' S, atrial strain; Aa' SR, atrial strain rate: **P**, paired *t *test; P*, analysis of variance; otherwise as in Table 3a.

**Aa' peak vel Low**: RA *vs *IAS, LA-Lat and LA-Ant (*P *< 0.001). No differences were found between any LA walls.

**Aa' peak vel Mid**: RA *vs *IAS, LA-Inf, LA-Post and LA-Ant *(P *< 0.001). No differences were found between any LA walls.

**Aa' disp. Low and Mid**: RA *vs *all walls (P < 0.001). No differences were found between any LA-walls.

**Aa' cont. Low and Mid**: No differences were found between walls

**Aa' SR**: RA *vs *all LA walls (*P *< 0.001), IAS *vs *LA-Lat and LA-Post (*P *< 0.001)

**Aa' S**: RA *vs *all LA walls (*P *< 0.001). No differences were found between any LA walls.

Tables 5 and 6 show the correlation coefficients between 2-D-derived and TVE-derived variables of LA and RA global mechanical function. There were no correlations between 2-D- and TVE-derived variables, apart from modest correlations between LA diameter and LA displacement, between RA long axis diameter and RA displacements, and between RA ejection fraction and strain rate.

**Table 5:** Correlation coefficients between 2-D- and TVE-derived variables of left global atrial mechanical function. Individual values of the inter-atrial septum, and the inferior, anterior, lateral and posterior LA walls were averaged.

	**LA-Aa' peak vel.**	**LA-Aa' disp.**	**LA-Aa' cont.**	**LA-Aa' SR**	**LA-Aa' S**
**LA-Diameter**	0.33	0.57*	0.43	0.2	0.21
**LA-Long axis**	0.00	0.17	0.02	-0.21	-0.08
**LA-Short axis**	-0.2	0.02	-0.08	-0.16	-0.07
**LA-Area**	-0.12	0.01	-0.13	-0.12	0.12
**LA-P-wave volume**	-0.15	0.02	-0.08	-0.14	-0.04
**LA-Maximal volume**	0.07	0.05	-0.1	-0.03	-0.21
**LA-Minimal volume**	0.2	0.15	0.13	-0.13	-0.21
**LA-Ejection fraction**	-0.27	-0.23	-0.31	-0.03	0.19
**LA-Active emptying**	-0.35	-0.22	-0.31	-0.03	0.19

Abbreviations: LA. Left atria; otherwise as in Table 3. * *P *< 0.01

**Table 6:** Correlation coefficients between 2-D- and TVE-derived variables of right global atrial mechanical function. Individual values of the inter-atrial septum, and the inferior, anterior, lateral and posterior LA walls were averaged.

	**RA-Aa' peak vel.**	**RA-Aa' disp.**	**RA-Aa' cont.**	**RA-Aa' SR**	**RA-Aa' S**
**RA-Long axis**	0.35	0.58*	0.53	0.26	-0.07
**RA-Short axis**	0.06	0.30	0.20	-0.00	-0,14
**RA-Area**	0.10	0.31	0.20	-0.00	-0.14
**RA-P-wave volume**	0.09	0.31	0.21	0.08	-0.15
**RA-Maximal volume**	-0.06	0.16	0.12	-0.04	-0.26
**RA-Minimal volume**	0.10	0.24	0.25	0.14	-0.18
**RA-Ejection fraction**	-0.33	-0.24	-0.35	-0.41*	-0.09
**RA-Active emptying**	0.07	0.27	0.12	-0.08	-0.08

Abbreviations: RA, right atrial; otherwise as in Table 3. * *P *< 0.01

The inter- and intra-observer measurement error and repeatability, as expressed by the British Standards Institution guidelines and coefficients of variation are presented in Table 7. The *PA-start interval *and the *PA-peak interval*, which mainly express atrial electrical function showed the largest inter- and intra-observer measurement errors and variability. However, the *A-wave duration *and the *total electromechanical activity*, which express a combination of the atrial electrical and mechanical functions, had better values of measurement error and repeatability. The same was true for all the TVE-derived variables that express regional and global atrial mechanical function.

**Table 7:** Assessment of inter- and intra-observer measurement error and repeatability according to the British Standards Institution guidelines and coefficients of variation

	**Inter-observer**		**Intra-observer**	
***Variable***	***BSI***	***CV (%)***	***BSI***	***CV (%)***

**P-Aa' start, ms**	37	24	28	19
**P-Aa' peak, ms**	51	16	47	14
**Aa' duration, ms**	32	8.8	25	7.5
**TEMA, ms**	53	9.7	45	8.2
**Aa' peak velocity, cm/s**	2.14	10.1	1.78	8.7
**Aa' displacement, mm**	1.81	12.3	1.77	11.8
**Aa' SR**	-0.79	9.4	-0.71	7.8
**Aa' S, %**	5.4	9.6	4.5	9.1
**LA maximal volume, mL**	22	18.4	18	14.7

Abbreviations: BSI, British Standards Institution; CV, coefficient of variation; otherwise as in Table 3.

## Discussion

The main new findings of this study of healthy young individuals are as follows. (1) Some TVE-derived variables indirectly reflect the atrial electrical activation that follows the known activation process as revealed by invasive electrophysiology. (2) The regional and global atrial mechanical function is explained by an upward movement of the atrial walls at the region near the A-V ring with a continuous reduction of this movement towards the upper levels of atrial walls. (3) The atrial mechanical function is quite similar in all LA walls; however, all indices of mechanical function were higher in the RA than in the LA. (4) There were no correlations between the 2-D- and TVE-derived variables expressing atrial mechanical function. (5) Values of measurement error and repeatability were good for atrial mechanical function, but only acceptable for electrical function.

Atrial electrical activation, as assessed by the *PA-start interval*, began at the RA and followed through the IAS, to the inferior and posterior LA walls. This is the known normal electrical activation process, as obtained by invasive electrophysiology techniques.[32,37] In the present study, there were no statistical significant differences in the *PA-start interval *between IAS and the inferior and posterior LA walls, indicating that the activation process could indistinctly occur through any of these walls, as demonstrated by the presence of preferential conduction pathways nearby the IAS, the posterior LA wall and the coronary sinus.[33,34,37]. In a recent study, using M-mode color tissue Doppler registrations of the tricuspide and mitral rings, an abnormal time interval from the onset of P wave until the backward motion of the left atrio-ventricular ring was used to indirectly detect abnormal atrial electromechanical coupling in patients with paroxysmal atrial fibrillation.[43]

The relation between atrial anatomy and its mechanical function has been poorly studied. The present study showed that all atrial walls actively moved upwards from the region of the A-V ring at late diastole, with a reduction of this movement towards the upper parts, thus empting the atria and contributing towards the last part of filling of the LV. This longitudinal movement of the atrial walls is probably related to the longitudinal endocardial muscular fibers along the walls of the LA and RA. The more pronounced longitudinal movement in the RA may be explained in part by the larger pectinate muscles in the RA, but also by the lower pressures in the heart's right side. To what extent circumferential contraction of the atrial muscle fibers might contribute to atrial mechanical function is unknown. Anatomically, the large amount of circumferential muscle fibers present in the vestibules of the RA and LA[30,31,44] might imply some kind of circumferential or radial contraction of the atria. However, no movement of the posterior LA wall at late diastole can be observed by conventional M-mode echocardiography. Other circumferential fibers, such as Bachman's bundle located at the subepicardium joining the RA and LA, seem to play a critical role for electrical impulse spreading[37] rather than in circumferential atrial contraction. The assessment of circumferential atrial mechanical function by conventional echocardiography and TVE remains elusive.

No correlations were found between 2-D- and TVE-derived variables of atrial mechanical function, as was also found in a previous study[29]. Although 2-D-derived variables measure volumes and volume-derived indices that might indicate some kind of atrial mechanical force, it was surprising to find no correlations between the variables obtained by the two different techniques. This might indicate that the velocities and the displacements registered from all atrial walls by TVE are less dependent on volume loading conditions than 2-D-derived variables and therefore could be used as reliable measurement of pure atrial mechanical contraction or inotropism. In fact, Donald et al. showed that LA function assessed by TVE was relatively independent of LV function.[45] It should also be considered that movements of the heart not related to atrial contraction might partly contribute to the velocities and displacements registered from all atrial walls. Therefore, 2-D- and TVE-derived variables might not be used interchangeably to assess atrial mechanical function.

Some measures of atrial electrical function, for example the *PA-start interval *and the *PA-peak interval*, had only fair measures of repeatability and measurement error. However, most of the TVE-derived variables expressing atrial mechanical function had good values of repeatability and measurement error. Assessing atrial mechanical function by measuring volumes is time-consuming and depends on age, gender, and body surface area[14,19] In addition, atrial volume indices are also dependent on loading conditions[46,47] and are not necessarily more reproducible than TVE-derived variables.

## Possible clinical implications

The identification of an abnormal electrical activation process could be of interest in some patients with atrial fibrillation or other supra-ventricular tachy-arrhythmias, in whom the premature atrial contraction acting as a triggering factor could be aggravated by local delayed conduction (reviewed in[48,49]). Further refinement of the TVE technique are necessary not only to identify the mechanical activation atrial sequence during normal sinus rhythm, but also to identify the origin and the activation sequence of supra-ventricular ectopic beats and in patients with RA, IAS or bi-atrial pacing. Thus, TVE could be an excellent adjunct to invasive electrophysiological techniques in selecting adequate patients and in the evaluation of atrial electromechanical consequences of RA, IAS or bi-atrial pacing.

The assessment of pure mechanical atrial function by means of atrial wall movements may give more concrete clues about the recovery process of atrial electromechanical function after conversion for atrial fibrillation and flutter and can give additional pathophysiological insights on the thromboembolic process that occur in some of those patients.[50] TVE-derived parameters may also give additional pathophysiological information on the process of atrial electromechanical remodeling that occurs in patients with sustained supra-ventricular tachy-arrhythmias.[51]

Several studies have shown the independent prognostic value of atrial function measurements in subsets of patients.[6,11,12] TVE-derived variables of atrial mechanical function may have an additional role for facilitating the assessment of atrial function and consequently in the process of risk stratification.

## Study limitations

The results of the present study refer only to a group of young healthy individuals and the values for each of the studied variables are, therefore, only applicable to that population group. As discussed, the measures of atrial electrical function showed only fair values of repeatability and measurement error. There were two reasons: the image acquisition rate (less than 100 frames per second) means an implicit measurement error of 10 ms; it was also difficult to identify the beginning of the P-wave in the ECG from the monitor in the echocardiography machine. Improving temporal resolution by image acquisition at more than 200 frames per second, and improving and adjusting the ECG quality in the present equipment may help solve or decrease this problem. The velocities and displacements registered by atrial walls do not only represent the process of atrial contraction, but also the translational movement of the heart. Until now, no appropriate algorithms that correctly deal with this problem have been found. Presently, it is not possible by means of TVE to simultaneously record the electromechanical function of all atrial walls in one heartbeat. The development of three-dimensional TVE may help resolve this difficulty.

## Conclusion

TVE is a noninvasive bedside tool that requires further refinements to provide reproducible, repeatable and potentially clinically useful data on atrial electromechanical function in health and disease.
